# The effect of *Tribulus terrestris* supplementation on inflammation, oxidative stress, and performance of recreational runners: study protocol for a randomized placebo-controlled trial

**DOI:** 10.1186/s13063-022-06630-0

**Published:** 2022-08-19

**Authors:** Marzieh Nejati, Parvin Dehghan, Mostafa Khani, Parvin Sarbakhsh

**Affiliations:** 1 Student Research Committee, Tabriz University of Medical Sciences, Tabriz, Iran; 2 Nutrition Research Center, Faculty of Nutrition and Food Science, Tabriz University of Medical Sciences, Tabriz, Iran; 3 Faculty of Physical Education and Sport Sciences, University of Tabriz, Tabriz, Iran; 4 Department of Statistics and Epidemiology, Tabriz University of Medical Sciences, Tabriz, Iran

**Keywords:** *Tribulus terrestris*, Recreational runners, EIOS, Oxidative stress, Inflammation, EIMD, Herbal supplement

## Abstract

**Background:**

High intensity and endurance exercises lead to exercise-induced oxidative stress (EIOS), exercise-induced muscle damage (EIMD), and inflammation, which are the influencing factors on muscle soreness, localized swelling, and sports performance decrease. Therefore, the purpose of this study is to determine the effectiveness of *Tribulus terrestris* (TT) as an herbal supplement with antioxidant and anti-inflammatory properties on the nutritional, oxidative, inflammatory, and anti-inflammatory status, as well as the sports performance of recreational runners.

**Methods/design:**

This study is a double-blind, randomized, placebo-controlled trial, which will be conducted among recreational runners of Tabriz stadiums, Iran. Thirty-four recreational runners will be selected, and participants will be assigned randomly to two groups: to receive 500 mg TT supplement or placebo capsules twice daily for 2 weeks. Both groups will do high-intensity interval training (HIIT) workouts during the study. Baseline and post-intervention body composition, muscle pain, and aerobic and anaerobic performance will be assessed. In addition, assessment of malondialdehyde (MDA), total antioxidant capacity (TAC), total oxidant status (TOS), superoxide dismutase (SOD), glutathione peroxidase (GPx), uric acid (UA), 8-iso-prostaglandin F2α (8-iso-PGF2α), protein carbonyl (PC), catalase (CAT), glutathione (GSH), nitric oxide (NO), high-sensitivity C-reactive protein (hs-CRP), interleukin-6 (IL-6), interleukin-10 (IL-10), tumor necrosis factor-alpha (TNF-α), creatine kinase (CK), myoglobin (MYO), lactate dehydrogenase (LDH), insulin-like growth factor-1 (IGF-1) irisin, cortisol, and brain-derived neurotrophic factor (BDNF) will be done during three blood samplings. Changes in oxidative stress, anti/inflammatory biomarkers, and sports performance will be assessed as primary outcomes.

**Discussion:**

This study will be the first to assess the potential effects of TT on recreational runners. Our results will contribute to the growing body of knowledge regarding TT supplementation on the nutritional, oxidative, inflammatory, and anti-inflammatory status and sports performance in recreational runners.

**Trial registration:**

Iranian Registry of Clinical Trials (www.irct.ir) (ID: IRCT20150205020965N8). Registration date: 13 February 2021.

**Supplementary Information:**

The online version contains supplementary material available at 10.1186/s13063-022-06630-0.

## Background

The World Health Organization indicates that regular physical activity is well-known for providing health benefits and preventing chronic and non-communicable diseases, including heart disease, stroke, diabetes, and several cancers [1]. Even so, several studies have proposed that intensive and unaccustomed exercises can lead to impaired muscle function, athletic performance, and recovery [2, 3]. High intensity and endurance exercises lead to an imbalance between oxidants and antioxidants in favor of the oxidants, defined as exercise-induced oxidative stress (EIOS) [4]. EIOS and the high level of reactive oxygen species can contribute to muscle damage [2, 4]. Besides, it should be noted that prolonged muscle contractions can result in a condition called exercise-induced muscle damage (EIMD). EIOS and EIMD are the influencing factors that result in muscle soreness, localized swelling, increased levels of creatine kinase (CK), lactate dehydrogenase (LDH), myoglobin (MYO), and inflammatory markers including C-reactive protein (CRP), interleukin 1 (IL-1), interleukin 6 (IL-6), and tumor necrosis factor-alpha (TNF-α) [3, 5],which lead to poor athletic performance. Considering the point that oxidative stress is the leading factor of the mentioned phenomenon, there is a growing interest in the use of antioxidant supplements by physically active individuals. Also, it seems that supplements with antioxidant and anti-inflammatory properties will provide more favorable effects. *Tribulus terrestris* (TT) is one of the herbal supplements with mentioned properties.

TT is a native plant of Iran classified in the family Zygophyllaceae [6, 7]. It can be found in a wide range of warm and humid regions such as the Mediterranean regions, Asia, Australia, Africa, and the warm areas of Europe [8] and contains a high concentration of active ingredients such as sterol saponins, flavonoids, tannins, terpenoids, phenol carboxylic acids, and alkaloids. The leaves, seeds, and fruits of TT are used for therapeutic purposes; however, studies indicate that the highest amount of TT's active ingredients is found in its fruit [6, 8]. According to existing studies, a variety of components in TT appear to contribute to its antioxidant capacity [9]. There may be a probable association between antioxidant activity and total saponin concentration of TT, according to Figueiredo *et al.’s* study [10]. Furthermore, Hammoda *et al.* found that the presence of di-p-coumaroylquinic acid derivatives would play a key role in the antioxidant activity of TT [11]. Ștefănescu *et al.* also credited TT’s antioxidant activity to its polyphenols and flavonoids [8]. Studies concentrating on the therapeutic effects of TT have assessed its potential effects on sexual enhancement [12], fertility [13], urinary tract stones [14, 15], diabetes [16, 17], cardiovascular diseases [18, 19], regarding its antioxidant [20, 21], and anti-inflammatory properties [22, 23]. Although a wide range of clinical studies has assessed the effects of TT on the enhancement of health [12–23], there are few randomized clinical trials on the effects of TT on athletes [24] and physically active individuals [25], and also, there is no randomized controlled trial on recreational runners. Therefore, this study has been designed to assess the efficacy of TT supplement on the nutritional, oxidative, inflammatory, and anti-inflammatory status and sports performance of recreational runners.

## Main aim

The present study is designed to determine the effect of TT supplementation on the nutritional, oxidative, inflammatory, and anti-inflammatory status and also sports performance of recreational runners.

### Primary objective

The primary objective is to assess the effect of TT supplementation on malondialdehyde (MDA), total antioxidant capacity (TAC), total oxidant status (TOS), superoxide dismutase (SOD), glutathione peroxidase (GPx), uric acid (UA), 8-iso-prostaglandin F2α (8-iso-PGF2α), protein carbonyl (PC), catalase (CAT), glutathione (GSH), nitric oxide (NO), hs-CRP, TNF-α, IL-6, IL-10, CK, LDH, MYO, muscle pain, and the aerobic and anaerobic performance of recreational runners with TT supplementation and high-intensity interval training (HIIT) training.

### Secondary objectives

The secondary objective is to assess the effect of TT supplementation on nutritional status (energy and macronutrient intake), insulin-like growth factor-1 (IGF-1), brain-derived neurotrophic factor (BDNF), irisin, cortisol, body mass index (BMI), and body composition of recreational runners with TT supplementation and HIIT training.

## Method

### Study design and setting

**Fig. 1:**
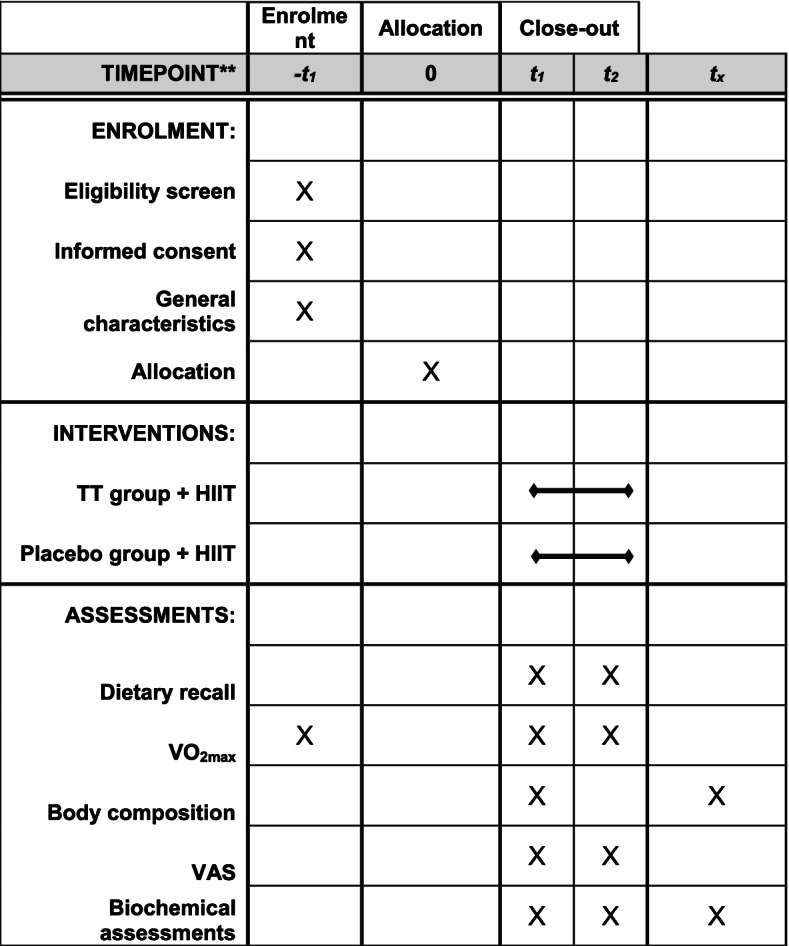
Study protocol diagram. TT, *Tribulus terrestris*; HIIT, high-intensity interval training; VAS, visual analog scale. -t1: 1 week pre-allocation, t0: allocation, t1: at the beginning of the study, t2: 2 weeks after the study beginning, and tx: 24 h after t2

**Fig. 2:**
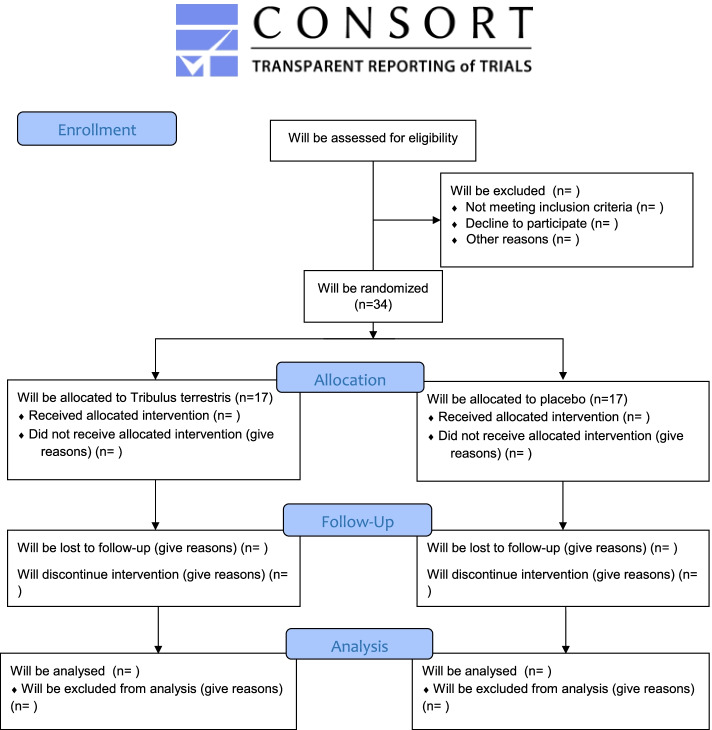
Flowchart of the trial

This study is a double-blind, randomized, placebo-controlled trial, which will be conducted among recreational runners of Tabriz stadiums, Iran. The study protocol followed the Standard Protocol Items: Recommendations for Clinical Interventional Trials (SPIRIT) guidelines (Additional file 1, SPIRIT Checklist), and the study protocol diagram is shown in Fig. 1 [26]. Subjects will undergo two body composition analyses and three times- blood samplings. Also, written informed consent will be obtained before initiating any research procedures. The flowchart of the trial is presented in Fig. 2. The research protocol is approved by the “Ethical Committee of the Tabriz University of Medical Sciences” and registered on the “Iranian Registry of Clinical Trials” website (www.irct.ir/, IRCT20150205020965N8) and is in compliance with the declaration of Helsinki ethical principles.

### Participants’ recruitment

The study population will include volunteer participants who have the eligible criteria. A total of 34 recreational runners (each group 17) will be recruited by advertisements from Tabriz stadiums, Iran. The volunteers will be screened by an initial face-to-face screening visit. In an organized meeting, participants will be provided with information, including the study procedure, requirements, possible risks, and benefits. A participant information sheet detailing study aims and requirements will be provided too.

### Eligibility

The volunteers will be included in the study if they (1) are healthy (confirmed by physical activity readiness questionnaire (PAR-Q) under the supervision of a physician [27]; (2) aged 18 to 35 years; (3) do running workouts for at least 3 days a week (240 min per week) during the last 2 years; (4) have a stable body mass during the last 5 months (changes less than 3 kg); (5) not receiving TT supplement, other antioxidants, and anti-inflammatory supplements in the last 3 months; (6) abstained from any high-intensity interval training during the last 3 months; and (7) are willing to cooperate in the study. Exclusion criteria include (1) musculoskeletal injuries; (2) smoking; (3) alcohol consumption; (4) hormone therapy; (5) long-term use of drugs and dietary supplements; (6) pregnancy; (7) lactation; (8) diabetes; (9) anemia (Hb <13g/dl); (10) cardiovascular disease; (11) infectious diseases; (12) malignancies; and (13) cognitive disorders.

### Sample size

The sample size was estimated using PASS software (version 15) based on CK changes as the primary outcome of the Ma *et al.* study. Considering the mean difference of 1128.4 units of enzyme activity per liter (U/L) between the two groups and also the SDs average of 871.05 U/L, the sample size was calculated [24]. A total of 17 recreational runners were calculated to be involved in each group with a 95% confidence level, a power of 90%, and considering the additional drop-out rate of 15%.

### Exercise protocol

This trial will use a HIIT exercise protocol as a high-intensity exercise activity designed for recreational runners using the American College of Sports Medicine (ACSM)’s physical activity recommendations [28]. The participants will do the HIIT program for two weeks (5 training sessions per week; a total of 10 sessions during the study period). A 15-min warm-up (with a variety of stretching, flexibility, walking, and running) will be included in each session. The main activity of both groups consists of two sessions with 3–4 repetitions and 30 s of running with an intensity of 90–100% of the heart rate reserve (HRR) (pressure perception 16–19) in each repetition. After each repetition and after each period, there will be 90–180 s and 2.5–4 min of active rest, respectively (active rest in the range of 40-50% of HRR) [29]. A 5-min cool-down at 45% HRR will end the training session.

### Supplementation protocol

Participants will consume the TT fruit supplement (TT, Dayan Pharma Co, Iran) or placebo (maltodextrin, Dayan Pharma Co, Iran) in random order. The supplement and placebo capsules will have perfectly the same shape, color, odor, and size. The company will code the supplement and placebo containers with different codes to keep participants and investigators blind until the end of the study. Both the TT and placebo capsules will be provided to participants weekly for two weeks. Subjects will be randomly assigned to two groups to receive 500 mg supplement or 500 mg placebo capsules, twice daily, for 2 weeks. Participants of both groups will be required to consume capsules twice daily (oral administration after breakfast and lunch) with a cup of water.

### Randomization and blinding

The recreational runners meeting the inclusion criteria will be randomized to receive either the TT (*n* = 17) or the maltodextrin capsules (*n* = 17). Randomizations will be conducted using random allocation software (RAS) via randomization blocks (Version 2.0, for Windows, Isfahan, Iran) [30]. After enrolling participants, they will be stratified into different blocks based on their VO_2max_ and gender distribution and then will be allocated to the intervention or control groups in random order. Using RAS, a random allocation sequence will be generated. Then, a third individual who is unaware of the study's aim will use computer-generated random sequences to allocate participants into TT and placebo groups.

### Adherence and compliance to the protocol of the study

There will be a one-week run-in period before study initiation. The study team will monitor participants daily during the trial, and any occurrence of adverse events will be reported. Participants will be asked to return any unused capsules to assess their level of compliance and adherence. Subjects will receive supplements every week and will be asked to bring all remaining ones of their last visit. If less than 90% of the capsules were used, the person would be excluded from the analysis. Besides, the adherence to the training program will be assessed by the number of sessions attended. If less than 90% of training sessions were attended each week, the person would be excluded.

### Questionnaires

Five types of questionnaires will be completed by each subject in this study: socio-demographic, 3-day food diary (2 weekdays and one weekend day) [31], the visual analog scale (VAS) [32], International Physical Activity Questionnaire (IPAQ) [33], and the PAR-Q [27]. Initially, PAR-Q will be used as a pre-study screening questionnaire that assesses a person’s eating habits, lifestyle, medical history, and physical fitness in several items; answering yes to any items of this questionnaire means that the participant will not enter into study [27]. The demographic data (age, gender, education, marital status, occupation, and income level) will also be collected. Runners will be asked to stick to their regular diet throughout the study. Assessments of dietary records at the beginning and end of the study will be done to ensure that the individuals followed their normal routine. Dietary intake of macronutrients and antioxidants (vitamin A, vitamin C, vitamin E, lycopene, β-carotene, β-cryptoxanthin, zinc, and selenium will be assessed using Nutritionist IV software (The Hearst Corp., San Bruno, CA, USA) [31]. The physical activity level of runners will also be assessed using the IPAQ questionnaire. Based on the IPAQ scoring system, the physical activity level of participants will be classified into three types: low activity, moderate activity, and high activity [33]. Also, a VAS will be used to determine the recreational runner’s muscle pain [32]. The VAS, a tool for reporting subjective muscle pain, has been used in several studies [34, 35]. It has also been validated as a measure for chronic and experimental pains [36]. Participants will rate the level of their pain on a 1–10 scale, from “no pain” to “extreme pain” [32].

## Outcome measurements

### Muscle pain assessment

A VAS will be used to determine the recreational runner’s muscle pain [32].

### Body composition analysis

Before and after the intervention, the body composition of participants will be measured using bioelectrical impedance analysis (Tanita BC-418, Tanita Corp., Tokyo, Japan).

### Anthropometric measurements

To measure the height and weight of participants, a stadiometer will be used (Seca, Hamburg, Germany). The body weight of participants will be measured with a minimum of wearing and without shoes to the nearest 100 g. Height will also be measured at a standing position and barefoot with an accuracy of 0.1 cm. BMI will finally be calculated using the measured weight and height. By dividing weight in kilograms by height squared in meters, BMI will be obtained [37]. Anthropometric measurements will be done twice, before and after the study.

### Aerobic, anaerobic performance

To assess the aerobic performance, the Cooper 12-min run test will be used. The runners will be asked to run as far as possible for 12 min. The covered distance then will be recorded [38]. Furthermore, the running-based anaerobic sprint test (RAST) will be used to assess the anaerobic performance of participants. RAST consists of six 35-m sprints separated by a 10-s recovery period. Each sprint will also be timed [39].

### Laboratory investigations

#### Blood sampling

Blood sampling will be done during three stages; before the intervention, immediately after the last training session on the fourteenth day, and 24 h after the previous training session. A 10-mL blood sample will be obtained during the three stages: before the intervention, immediately after the last training session on the fourteenth day, and 24 h after the last training session by a lab technician. Plasma centrifugation will be carried out at a speed of 1500 RPM for 20 min. Then, the plasma will be collected into separate micro-tubes and placed in a freezer at a temperature of − 70 °C before laboratory analysis.

#### Protocol amendments

Any changes or amendments in protocol will be reviewed by the principal investigator and approved by the other study investigators. Any modifications will be eventually reported.

#### Data collection and management

MN will collect data from questionnaires. After completion of the questionnaires, MN will assess the collected data and, if there were any discrepancies in answers, the questioner will be requested to answer more clearly to reduce bias.

#### Statistical analysis

Statistical analysis will be done using SPSS version-24 software (SPSS, Inc. Chicago, IL, USA). Regarding the missing values, the data analysis will be subjected to intention-to-treat (ITT) analysis. We will use the multiple imputation approach to perform ITT. The Kolmogorov-Smirnov test and Q–Q plot will be used to examine the normal distribution of variables. In order to compare the baseline value of quantitative and qualitative variables, the *t*-test and chi-square test will be used. To determine the change of variables at baseline and after the intervention, the paired sample *t*-test will be conducted. The mean change of the placebo and intervention group variables will also be compared using an analysis of covariances after adjusting the confounding variables. Mann-Whitney test will be used for quantitative non-normal variables. In order to compare the mean changes of the baseline and end of the trial, the paired-sample *t*-test and Wilcoxon test will be used for normal and non-normal data, respectively. Regarding repeated measured variables (three-time measurements), a two-way analysis of variance (ANOVA) with the Sidak post-hoc test will be applied to assess the time × group interactions. Also, the Mauchly sphericity test will be used to determine data homogeneity; in presence of a violation, the Greenhouse-Geisser adjustment will be used. *P* < 0.05 will be considered statistically significant in all tests. Furthermore, it is notable that in the case of non-normally distributed data, we will do Log transformations.

#### Ancillary and post-trial care

The trial is not expected to cause any specific side effects. All participants will be guided to make informed decisions about the supplement intake during high-intensity exercise training.

## Discussion

The interest in natural supplements to promote athletic performance has increased these days among athletes. Recently, TT supplementation has also been raised in various exercises due to its beneficial consequence, which was seen in strength athletes [24]. In a study on boxers, conducted by Ma *et al.*, it has been indicated that TT lowered IGF-1 binding protein-3 levels, which may improve IGF-1 bioactivity, as well as increasing muscle strength and decreasing post-exercise CK levels when used in conjunction with training [24]. In a recent study pilot study also, Talemi *et al.* showed that TT might be effective in the reduction of CK and LDH following the high-intensity resistance exercise in non-athletes [40]. According to a review done by Zhu *et al.*, it has been shown that the TT has a wide range of healthful properties, including antioxidant activity, anti-inflammatory activity, antitumor activity, antibacterial activity, hepatoprotective activity, anthelmintic and larvicidal activity, anticaries activity, antiaging and memory improvement activity [41]. Also, it is worth mentioning that the chemical constituents of TT including steroidal saponins and flavonoids, which have prominent anti-inflammatory and antiaging activities, were found to be the key contributors to conventional pharmacological activities of TT [41]. TT can promote athletic performance and decrease fatigue due to its antioxidant activity and anti-inflammatory activity; in addition, it has been shown that TT supplementation may result in decreased IGF-1 binding protein-3 levels and increased IGF-1 bioactivity. IGF-1 is the upregulator of muscle growth and muscle strength which leads to muscle hypertrophy, repair of muscle damage, and eventually the promoted athletic performance [24, 42, 43].

To the best of our knowledge, this study will be the first to assess the potential effects of TT on recreational runners. The present protocol is based on a randomized placebo-controlled trial design, which presents the strongest empirical evidence regarding the TT supplementation and determines the causality. Besides, the control over participants’ diet and repeated blood collection in this study will provide more precise details.

It also should be noted that this is a short-term study that will necessitate additional research in the long run. Due to financial constraints, we did not analyze the other inflammatory and oxidative parameters. It is also notable that the study participants did not undergo a specialized diet. Furthermore, our protocol is designed for recreational runners and cannot be applied to elite runners.

## Trial status

The present protocol is version 1, dated 25 January 2021. The trial has not yet begun, and the process of recruiting participants is ongoing.

## Supplementary Information

**Additional file 1.** Standard Protocol Items: Recommendations for Interventional Trials (SPIRIT) 2013 Checklist.

## Data Availability

By the end of the trial, all primary and secondary outcome data will be published; thus, the participant-level data will be available from the corresponding author on reasonable request.

## Abbreviations

TT: *Tribulus terrestris*
EIOS: Exercise-induced oxidative stress
EIMD: Exercise-induced muscle damage
HIIT: High-intensity interval training
ACSM: American College of Sports Medicine
BMI: Body mass index
MDA: Malondialdehyde
TAC: Total antioxidant capacity
OSI: Oxidative stress index
TOS: Total oxidant status
SOD: Superoxide dismutase
GPx: Glutathione peroxidase
CAT: Catalase
UA: Uric acid
8-iso-PGF2α: 8-Iso-prostaglandin F2α
PC: Protein carbonyl
hs-CRP: High-sensitivity C-reactive protein
IL-6: Interleukin-6
IL-10: Interleukin-10
CK: Creatine kinase
LDH: Lactate dehydrogenase
MYO: Myoglobin
TNF-α: Tumor necrosis factor-alpha
IGF-1: Insulin-like growth factor-1
GSH: Glutathione
NO: Nitric oxide
BDNF: Brain-derived neurotrophic factor
HRR: Heart rate reserve
SPIRIT: Standard protocol items: recommendations for clinical interventional trials
VAS: Visual analog scale
PAR-Q: Physical activity readiness questionnaires
IPAQ: International physical activity questionnaire
RAST: Running-based anaerobic sprint test
